# The pretreatment erythrocyte sedimentation rate predicts survival outcomes after surgery and adjuvant radiotherapy for extremity soft tissue sarcoma

**DOI:** 10.1186/s13014-019-1331-z

**Published:** 2019-07-04

**Authors:** Geumju Park, Si Yeol Song, Jin-Hee Ahn, Wan-lim Kim, Jong-seok Lee, Seong-Yun Jeong, Jae Won Park, Eun Kyung Choi, Wonsik Choi, In-Hye Jung

**Affiliations:** 1 Department of Radiation Oncology, Inje University Haeundae Paik Hospital, Busan, South Korea; 2 Department of Radiation Oncology, Asan Medical Center, University of Ulsan College of Medicine, 88, Olympic-ro 43-gil, Songpa-gu, Seoul, 05505 South Korea; 3 Department of Internal Medicine, Asan Medical Center, University of Ulsan College of Medicine, Seoul, South Korea; 4 Department of Orthopedic Surgery, Asan Medical Center, University of Ulsan College of Medicine, Seoul, South Korea; 5 Asan Institute for Life Science, Asan Medical Center, University of Ulsan College of Medicine, Seoul, South Korea; 6 Department of Radiation Oncology, Yeungnam University Medical Center, Daegu, South Korea; 7 Department of Radiation Oncology, Gangneung Asan Hospital, University of Ulsan College of Medicine, Gangneung, South Korea

**Keywords:** Soft tissue sarcoma, Sarcoma, Erythrocyte sedimentation rate, ESR, Biomarker, Prognostic factor

## Abstract

**Background:**

Systemic inflammation plays a critical role in cancer progression and oncologic outcomes in cancer patients. We investigated whether preoperative inflammatory biomarkers, including C-reactive protein (CRP), erythrocyte sedimentation rate (ESR), and neutrophil to lymphocyte ratio (NLR), could be surrogate biomarkers for predicting overall survival (OS) in soft tissue sarcoma (STS) patients treated with surgery and postoperative radiotherapy.

**Methods:**

A series of 99 patients who presented with localized extremity STS were retrospectively reviewed. The preoperative CRP levels, ESR, and NLR were evaluated for associations with OS, disease-free survival (DFS), local recurrence-free survival (LRFS), and distant metastasis-free survival (DMFS). Cutoff values for CRP, ESR, and NLR were derived from receiver-operating characteristic curve analysis.

**Results:**

Elevated CRP (> 0.14 mg/dL), ESR (> 15 mm/h), and NLR (> 1.95) levels were seen in 33, 44, and 45 patients, respectively. Of these three inflammatory biomarkers, elevated CRP and ESR were associated with a poorer OS (CRP: *P* = 0.050; ESR: *P* = 0.001), DFS (CRP: *P* = 0.023; ESR: *P* = 0.003), and DMFS (CRP: *P* = 0.015; ESR: *P* = 0.001). By multivariate analysis, an elevated ESR was found to be an independent prognostic factor for OS (HR 3.580, *P* = 0.025) and DMFS (HR 3.850, *P* = 0.036) after adjustment for other established prognostic factors.

**Conclusions:**

The preoperative ESR level is a simple and useful surrogate biomarker for predicting survival outcomes in STS patients and might improve the identification of high-risk patients of tumor relapse in clinical practice.

## Background

Soft tissue sarcoma (STS) denotes a rare group of cancers that arises in mesenchymal cells and accounts for less than 1% of all malignancies. STS represents heterogeneous histologic subtypes, anatomic sites, and aggressiveness, with wide variability in clinical outcomes that has made its prognosis difficult. Therefore, identification of prognostic factors is essential for selecting STS patients at risk of a poor prognosis and thus delivering more intensive adjuvant therapy and surveillance. Currently accepted, relevant prognostic parameters for STS include age at diagnosis, histologic grade, tumor size, and tumor depth [1, 2]. Although the standard treatment of localized STS is conservative surgery with wide excision whenever possible plus adjuvant radiotherapy (RT), more than 30% of these patients develop metastases after curative surgery, and approximately 50% of the cases who present with high-risk STS die of this disease [3]. Hence, there is an urgent need for more readily available molecular biomarkers to improve the identification of STS patients at high risk of tumor relapse.

It is now evident that inflammatory responses play a critical role in tumor development, and some of the underlying molecular mechanisms have been elucidated. Several pro-inflammatory gene products have been identified that are closely linked with various steps of tumorigenesis, including cellular transformation, promotion, survival, proliferation, invasion, angiogenesis, and metastasis [4, 5]. It is also now well recognized that chronic inflammation is a risk factor for most types of cancer. Thus, inflammatory biomarkers might be used to predict cancer aggressiveness and monitor the progression.

C-reactive protein (CRP) levels and the erythrocyte sedimentation rate (ESR) are non-specific but commonly used laboratory markers of the systemic inflammatory response. CRP is a major acute phase reactant that is produced in hepatocytes in response to inflammation, infection, and malignancy. The ESR is a hematological test routinely used as an indirect parameter of increased acute phase reactants, particularly fibrinogen. Increased preoperative serum CRP levels have been associated with poor survival in many cancers, including colorectal cancer, renal cell carcinoma, breast cancer, and non-small cell lung cancer [6–9]. Similarly, several clinical studies have shown that a high ESR indicates a poor prognosis in renal cell carcinoma and multiple myeloma [10, 11]. Systemic inflammation also causes changes in the white blood cell (WBC) count, including neutrophils and lymphocytes. However, the WBC level fluctuates on a daily basis. The neutrophil to lymphocyte ratio (NLR) can more accurately indicate the inflammation status of a patient. In vitro studies have suggested that a high neutrophil count suppresses the antitumor efficacy of the host immune system [12]. Recently, a high NLR has been reported as a prognostic marker in patients with non-small cell lung cancer, cervical carcinoma, hepatocellular carcinoma, gastric cancer, and metastatic brain tumor [13–18].

There have been few reports regarding the prognostic value of preoperative CRP levels, the ESR, and the NLR in patients with STS [19–23]. In addition, most of the existing studies reviewed heterogeneous groups, included various sites and treatment modalities. Therefore, in our current study, we assessed whether the preoperative serum CRP levels, the ESR, and the NLR could act as surrogate biomarkers for predicting overall survival (OS) in extremity STS patients treated with surgery and postoperative RT.

## Methods

Institutional review board of Asan Medical Center (AMC-IRB) approved this retrospective study (2016–0447). We retrospectively reviewed the medical records of 99 patients who were treated with surgery and postoperative RT for primary localized STS of extremity between 2001 and 2013 at our institution.

Histological subtypes were classified using the World Health Organization classification (2002) for STS. Tumors were graded according to the French Fédération Nationale des Centres de Lutte Contre le Cancer (FNCLCC) grading system [24]. Serum CRP levels, the ESR, WBC counts (including neutrophils and lymphocytes), and hemoglobin (Hb) levels were obtained 1–7 days before surgery. In patients treated with neoadjuvant chemotherapy, inflammatory markers were measured prior to chemotherapy. Normal serum CRP levels and ESRs are ≤0.6 mg/dL and ≤ 20 mm/h at our institution, respectively. The NLR was defined as the absolute neutrophil count divided by the absolute lymphocyte count. Anemia was defined as Hb levels below 12 g/dL.

All patients were treated with limb-sparing surgery and postoperative external-beam RT. If patients had gross residual disease after a non-anatomical operation, a second oncologic re-excision was recommended. General indications for postoperative RT at our institution included high-grade, large tumors, and close or positive resection margins. All patients were immobilized using custom-made molds and underwent CT simulation for treatment. The clinical target volume (CTV) encompassed the entire involved compartment, operative bed, incision site, and drain site. An additional longitudinal margin of 5 cm and a radial margin of 1.5–2.0 cm were generally added to the CTV. The planning target volume was expanded by 0.7–1.0 cm from the CTV. The RT dose was 45–50 Gy with the initial plan, which was then boosted to 60–66 Gy with a fraction size of 1.8 or 2 Gy.

Patients were followed up regularly by physical examination at 3-month intervals for the first 2 years and then at 6-month interval thereafter. Occasionally, magnetic resonance imaging or ultrasonography was performed if the symptoms were aggravated. OS, disease-free survival (DFS), local recurrence-free survival (LRFS), and distant metastasis-free survival (DMFS) were estimated from the date of surgery to the date of death, last follow-up, or tumor recurrence using the Kaplan-Meier method. A Kaplan-Meier plot with a log-rank test was used for univariate analysis to determine factors predictive of survival. Prognostic factors with a *P* value ≤0.05 in univariate analysis were evaluated by multivariate analysis using the Cox proportional hazard model. The Chi-square analysis was used to determine if there was a relationship between inflammatory markers and chronic comorbidity. Receiver-operating characteristic (ROC) curve analysis was employed to determine optimal cutoff values for CRP level, the ESR, and the NLR. *P* values ≤0.05 were considered statistically significant. All statistical analyses were performed using SPSS software (version 12.0; SPSS, Inc., Chicago, IL).

## Results

Patient characteristics are listed in Table 1. Of the 99 patients in our current study series, 51.5% were male with a median age of 50 years (range, 15–84 years). Twenty-seven patients had chronic comorbidity, but none showed active inflammatory condition. Seventy-three of the tumors in our study cohort were located in a lower extremity, most often in the thigh. The median tumor size was 7 cm (range, 0.7–25 cm). Sixty-nine of these tumors were anatomically located at a deep depth, either involving the superficial fascia or locating beneath the fascia. Fifty patients were histologically classified as grade 2 according to the FNCLCC grading system, 32 patients had grade 3 tumors, and 17 patients had grade 1 tumors. The most frequent histological subtype was malignant fibrous histiocytoma (MFH, 35.4%), followed by myxoid liposarcoma (26.3%) and synovial sarcoma (10.1%). Close or positive resection margin status was found in 19 patients. Systemic chemotherapy was delivered to 41 patients: 36 patients received adjuvant chemotherapy and 5 patients received both neoadjuvant and adjuvant chemotherapy. Most patients received a combination regimen of doxorubicin, cyclophosphamide, vincristine, and dacarbazine, with a median of 4 cycles. The median RT dose was 60 Gy (range, 46–66 Gy).

**Table 1:** Patient characteristics

	No. of patients (%)
Age (years)	
Median	50
Range	15–84
Gender	
Male	51 (51.5)
Female	48 (48.5)
Any chronic comorbidity	
No	72 (72.7)
Yes	27 (27.3)
Tumor site	
Lower extremity	73 (73.7)
Upper extremity	26 (26.3)
Tumor size (cm)	
≤ 5	34 (34.3)
> 5	61 (61.6)
Unknown^a^	4 (4.0)
Tumor depth	
Superficial	30 (30.3)
Deep	69 (69.7)
FNCLCC grade	
1	17 (17.2)
2	50 (50.5)
3	32 (32.3)
Histological subtype	
MFH	35 (35.4)
Myxoid liposarcoma	26 (26.3)
Synovial sarcoma	10 (10.1)
Dedifferentiated liposarcoma	8 (8.1)
Myxofibrosarcoma	5 (5.1)
Fibrosarcoma	5 (5.1)
MPNST	5 (5.1)
Ewing sarcoma/ PNET	5 (5.1)
Resection margin	
Negative	77 (77.8)
Close (≤2 mm)	9 (9.1)
Positive	10 (10.1)
Unknown^a^	3 (3.0)
Treatment modality	
Surgery + RT	58 (58.6)
Surgery + RT + CXT	41 (41.4)
RT dose (Gy)	
Median	60
Range	46–66

*Abbreviations*: *FNCLCC* Fédération Nationale des Centres de Lutte Contre le Cancer, *MFH* malignant fibrous histiocytoma, *MPNST* malignant peripheral nerve sheath tumor, *PNET* primitive neuroectodermal tumor, *RT* radiotherapy, *CXT* chemotherapy

^a^It was not possible to evaluate in patients who underwent excision at an outside institution

Preoperative laboratory results are shown in Table 2. Anemia was seen in 15 patients. Elevated CRP level (> 0.6 mg/dL) and an elevated ESR (> 20 mm/h) were observed in 9 and 36 cases, respectively. The median value for NLR was 1.84 (range, 0.41–11.68). Cutoff values on ROC curve analysis were 0.14 mg/dL for CRP, 15 mm/h for ESR, and 1.95 for NLR, respectively.

**Table 2:** Details of preoperative laboratory results of 99 patients with extremity soft tissue sarcoma

	No. of patients (%)
Hemoglobin (g/dL)	
≥ 12	84 (84.8)
< 12	15 (15.2)
CRP (mg/dL)	
≤ 0.6	85 (85.9)
> 0.6	9 (9.1)
Unknown	5 (5.1)
ESR (mm/h)	
≤ 20	55 (55.6)
> 20	36 (36.4)
Unknown	8 (8.1)
NLR	
Median	1.84
Range	0.41–11.68
Unknown	2 (2.0)

*Abbreviations*: *CRP* C-reactive protein, *ESR* erythrocyte sedimentation rate, *NLR* neutrophil to lymphocyte ratio

**Fig. 1:**
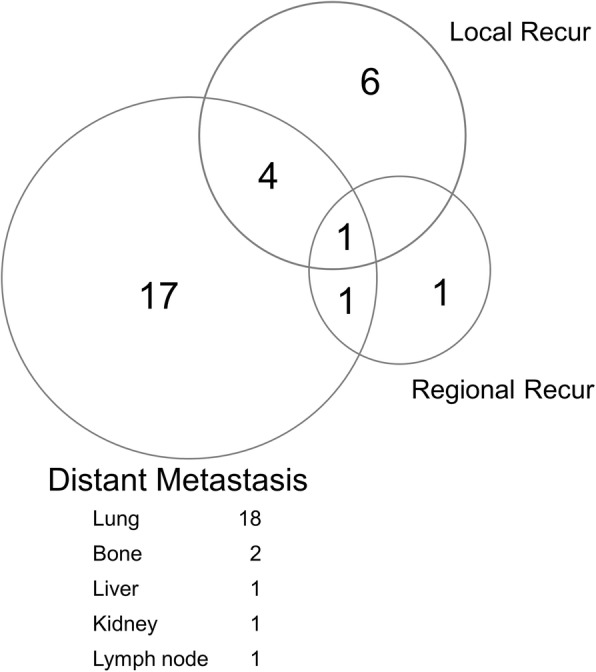
Patterns of failure in 99 patients with extremity soft tissue sarcoma

Among the 99 patients in our current study cohort, 30 patients presented with recurrence during the follow-up period. Figure 1 shows the patterns of these recurrence sites. Distant metastasis was the most common failure (23 patients) and most frequently occurred in the lungs (18 patients), followed by bone (two patients), liver (one patient), kidney (one patient), and lymph nodes (one patient). Six patients had an isolated local recurrence, whereas one patient had an isolated regional recurrence. Of the 11 patients with local recurrence, eight failed within the RT field, three failed at the margin, and zero had an out-of-field failure. The median interval from surgery to the development of recurrence was 12.8 months (range, 2.8–69.2 months).

The median follow-up time was 85.2 months (range, 10.4–195.8 months). At the time of analysis, 26 patients had died. The most common cause of death was progression of a distant metastasis (22 patients). Two of the remaining patients died of locoregional recurrence, and the other two died of other medical conditions. The OS, DFS, LRFS, and DMFS at 5 years were 77.0, 69.8, 88.2, and 77.1%, respectively.

In univariate analysis (Table 3), factors significantly associated with a favorable OS were an age ≤ 60 years (85.9% vs. 52.0% at 5 years, *P* < 0.001), FNCLCC grade 1–2 (84.8% vs. 60.2% at 5 years, *P* = 0.012), CRP level ≤ 0.14 mg/dL (84.5% vs. 69.3% at 5 years, *P* = 0.050), and an ESR ≤ 15 mm/h (91.2% vs. 69.4% at 5 years, *P* = 0.001). For DFS, age (*P* = 0.001), FNCLCC grade (*P* < 0.001), CRP (*P* = 0.023), and ESR (*P* = 0.003) were significant prognostic factors. For LRFS, FNCLCC grade (*P* = 0.008), resection margin (*P* = 0.001), and ESR (*P* = 0.035) were significant prognostic factors. Age (*P* = 0.002), FNCLCC grade (*P* = 0.003), CRP (*P* = 0.015), and ESR (*P* = 0.001) were significantly associated with DMFS.

**Table 3:** Univariate survival analysis of 99 patients with extremity soft tissue sarcoma

	No.	5-year OS		5-year DFS		5-year LRFS		5-year DMFS	
		%	*P* value	%	*P* value	%	*P* value	%	*P* value
Age (years)									
≤ 60	74	85.9	< 0.001	77.4	0.001	89.4	0.236	84.4	0.002
> 60	25	52.0		43.2		83.6		55.7	
Tumor size (cm)									
≤ 5	34	87.5	0.130	78.0	0.229	96.7	0.162	84.2	0.263
> 5	61	69.8		63.5		82.3		71.8	
Tumor depth									
Superficial	30	89.3	0.205	79.0	0.300	92.6	0.734	86.2	0.296
Deep	69	71.9		65.8		86.2		73.2	
FNCLCC grade									
1–2	67	84.8	0.012	81.5	< 0.001	93.4	0.008	84.5	0.003
3	32	60.2		44.8		75.3		61.4	
Resection margin									
Negative	77	80.0	0.065	73.6	0.109	93.9	0.001	78.9	0.202
Close or Positive	19	67.4		52.6		65.1		68.0	
Chemotherapy									
No	58	80.2	0.522	73.0	0.535	86.0	0.313	84.0	0.115
Yes	41	72.7		65.5		91.5		67.9	
Hemoglobin (g/dL)									
≥ 12	84	76.7	0.914	68.0	0.398	87.5	0.574	76.6	0.765
< 12	15	79.4		80.0		93.3		80.0	
CRP (mg/dL)									
≤ 0.14	61	84.5	0.050	79.3	0.023	92.6	0.376	84.5	0.015
> 0.14	33	69.3		57.1		84.1		66.5	
ESR (mm/h)									
≤ 15	47	91.2	0.001	89.1	0.003	97.7	0.035	93.5	0.001
> 15	44	69.4		57.8		80.6		67.4	
NLR									
≤ 1.95	52	84.0	0.054	78.4	0.174	89.3	0.793	84.4	0.170
> 1.95	45	70.2		63.0		89.0		69.9	

*Abbreviations*: *OS* overall survival, *DFS* disease-free survival, *DMFS* distant metastasis-free survival, *LRFS* local recurrence-free survival, *FNCLCC* Fédération Nationale des Centres de Lutte Contre le Cancer, *CRP* C-reactive protein, *ESR* erythrocyte sedimentation rate, *NLR* neutrophil to lymphocyte ratio

**Fig. 2:**
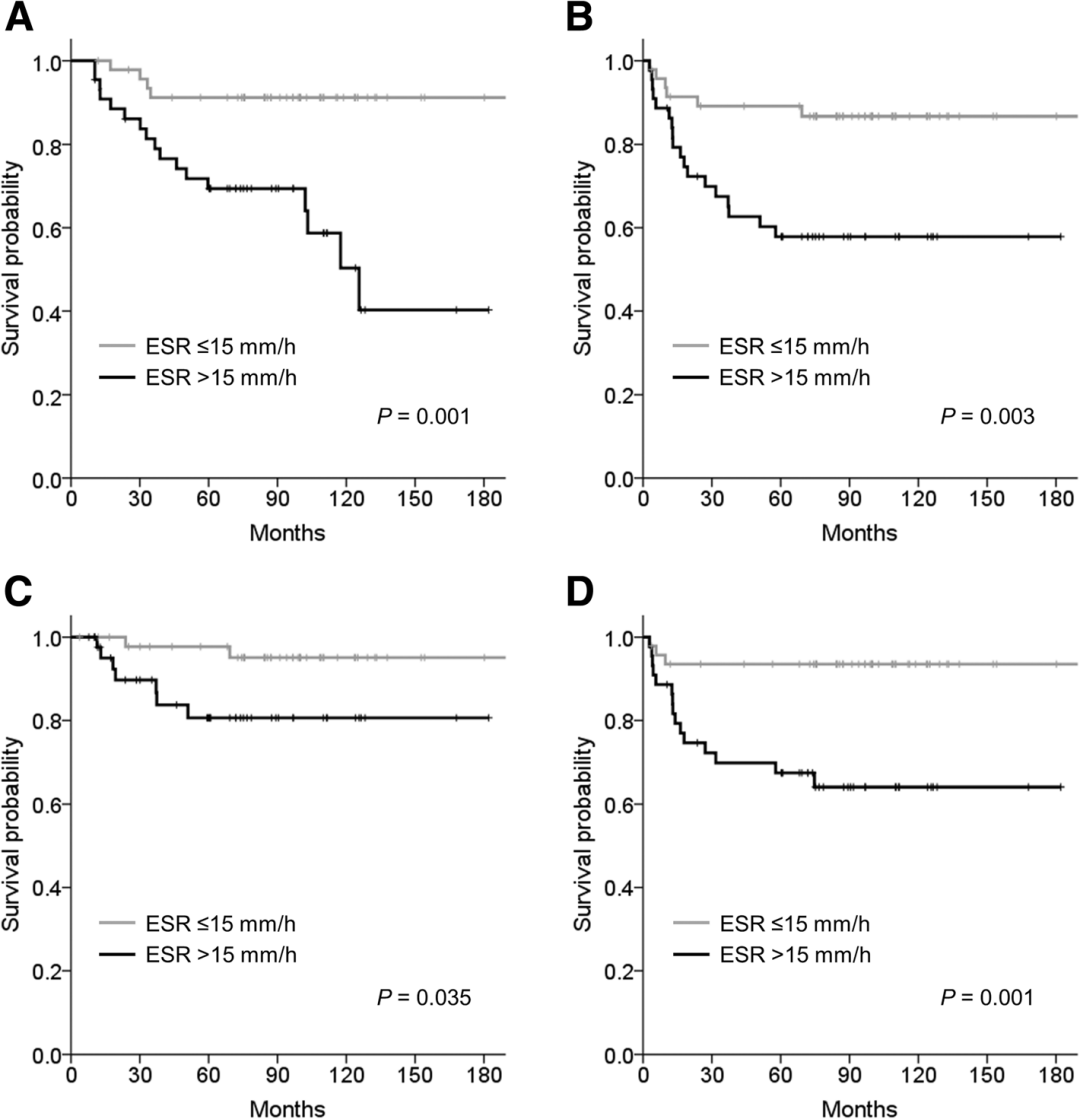
Kaplan-Meier survival curves stratified by erythrocyte sedimentation rate (ESR) status: (**a**) overall survival; (**b**) disease-free survival; (**c**) local recurrence-free survival; (**d**) distant metastasis-free survival

In multivariate analysis (Table 4), an elevated ESR was found to be an independent prognostic factor for OS (HR 3.580, *P* = 0.025) and DMFS (HR 3.850, *P* = 0.036), and had borderline statistical significance for DFS (HR 2.327, *P* = 0.079) and for LRFS (HR 3.947, *P* = 0.090). Figure 2 shows the Kaplan-Meier survival curves stratified by ESR status. The Chi-square analysis showed that there was no significant relationship between levels of inflammatory markers and chronic comorbidity (CRP, *P* = 0.115; ESR, *P* = 0.171; NLR, *P* = 0.373).

**Table 4:** Multivariate survival analysis of 99 patients with extremity soft tissue sarcoma

		OS		DFS		DMFS	
		Hazard Ratio (95% CI)	*P* value	Hazard Ratio (95% CI)	*P* value	Hazard Ratio (95% CI)	*P* value
CRP	CRP > 0.14 mg/dL	1.594 (0.685–3.711)	0.279	1.532 (0.702–3.346)	0.284	1.935 (0.789–4.744)	0.149
	Age > 60	3.315 (1.430–7.685)	0.005	2.377 (1.093–5.171)	0.029	2.811 (1.173–6.739)	0.021
	FNCLCC grade 3	2.119 (0.902–4.977)	0.085	3.849 (1.702–8.704)	0.001	2.496 (1.015–6.137)	0.046
ESR	ESR > 15 mm/h	3.580 (1.174–10.916)	0.025	2.327 (0.908–5.963)	0.079	3.850 (1.090–13.592)	0.036
	Age > 60	2.677 (1.104–6.491)	0.029	2.325 (1.028–5.260)	0.043	2.642 (1.033–6.754)	0.043
	FNCLCC grade 3	2.690 (1.123–6.447)	0.026	5.292 (2.158–12.978)	< 0.001	3.689 (1.372–9.918)	0.010

*Abbreviations*: *OS* overall survival, *DFS* disease-free survival, *DMFS* distant metastasis-free survival, *CI* confidence interval, *CRP* C-reactive protein, *FNCLCC* Fédération Nationale des Centres de Lutte Contre le Cancer, *ESR* erythrocyte sedimentation rate

## Discussion

In our present study, an elevated preoperative ESR was found to be an independent prognostic factor for OS and DMFS in localized extremity STS patients. Literature regarding the prognostic ability of the serum ESR on survival in STS patients is sparse. Choi et al. demonstrated that the CRP and ESR level were associated with disease-specific survival in 162 STS patients [21]. The major difference of this current study from the paper by Choi et al. is the proportion of patients received postoperative RT (100% vs. only 44% in Choi et al.) in patient characteristics. In result, Choi et al. asserted that elevation of multiple inflammatory markers was a stronger prognostic factor although NLR failed to show its significance unlike CRP or ESR in multivariate analysis. However, preoperative ESR was a unique marker for survival in this current study. Several clinical studies in other cancer patients have shown that an elevated ESR is associated with a poor prognosis. In other previous reports, an elevated ESR suggested the aggressive disease and poor survival outcomes after surgical treatment in renal cell carcinoma [10, 25]. Also, an association between an elevated ESR and poor oncologic outcomes has been reported in multiple myeloma and Hodgkin’s disease [11, 26].

The ESR is one of the most commonly used and inexpensive markers for systemic inflammation in clinical practice. However, the molecular and cellular mechanisms underlying the relationship between the ESR and poor oncologic outcomes remain poorly understood. Recently, the tumor-induced inflammatory response has been described as one of the key events in cancer development and progression [4, 5, 27]. Inflammation can affect the tumor microenvironment, including growth factors that sustain proliferative signaling, survival factors that limit cell death, and proangiogenic factors and extracellular matrix-modifying enzymes that facilitate angiogenesis, invasion and metastasis [27]. In previous studies, a possible correlation between the inflammatory cytokine interleukin-6 (IL-6) and CRP was suggested [28, 29]. In the tumor microenvironment, IL-6 can be produced in response to tumor cells, tissue necrosis, and tissue inflammation. It has also been reported that the production of CRP in hepatocytes is stimulated by IL-6 [30, 31]. Although the ESR level is thought to correlate with tumor burden and histologic grade, and to be affected by anemia, the pathways that lead to an elevated ESR in cancer patients require further elucidation [21, 32, 33].

There have been several studies that evaluated the prognostic power of other inflammatory markers, such as CRP levels and the NLR in non-metastatic STS patients (Table 5). Nakamura et al. reported that pretreatment CRP levels were a prognostic factor for DFS [19]. Szkandera et al. determined that increased CRP levels were significantly associated with survival outcomes [20]. The ability of the Kattan nomogram [34] to predict sarcoma-specific death was improved when serum CRP was added to established prognostic factors such as age, tumor size, histological grade, histological subtype, tumor depth, and tumor site. Recent data have also indicated that a high NLR might be a predictor of mortality in patients with STS [22, 23]. Recently, the NLR was shown to be a possible prognostic factor for survival in metastatic STS patients [35]. However, the CRP or NLR level was not associated with survival in this study. Cut-off values of CRP and NLR were 0.14 mg/dL and 1.95 respectively which are lower value than those of other studies [19–23]. We guessed the low pretreatment baseline level of CRP or NLR was a reason why these markers could not reach significant prognostic factor for survival or metastasis. We are planning to investigate why there are discordances between inflammatory markers such as CRP, ESR, NLR or others in oncologic conditions.

**Table 5:** Literature review of significant inflammatory prognostic factors for overall survival or disease-free survival on multivariate analysis

Study (Ref.)	No.	Site	Treatment (No.)	Prognostic factors
Nakamura [19]	102	All	OP (100) or RT (2) ± CXT (15)	CRP (≤0.3 vs. > 0.3 mg/dL)
Szkandera [20]	304	All	OP (111) or OP + RT (193) ± CXT (39)	CRP (< 0.69 vs. ≥0.69 mg/dL)
Choi [21]	162	Extremity	OP (90) or OP + RT (72) ± CXT (43)	ESR (≤10 vs. > 10 mm/h)
				CRP (≤0.2 vs. > 0.2 mg/dL)
Szkandera [22]	260	All	OP (93) or OP + RT (167) ± CXT (35)	NLR (< 3.58 vs. ≥3.58)
Idowu [23]	83	All	OP (55) or OP + RT (28) ± CXT (6)	NLR (< 5 vs. ≥5)
This study	99	Extremity	OP + RT (58) ± CXT (41)	ESR (≤15 vs. > 15 mm/h)

*Abbreviations*: *OP* operation, *RT* radiotherapy, *CXT* chemotherapy, *CRP* C-reactive protein, *ESR* erythrocyte sedimentation rate, *NLR* neutrophil to lymphocyte ratio

We should consider how we can overcome poor survival and increased incidence of distant metastasis resulted from the elevated pretreatment ESR. The most probable suggestion in clinic would be concurrent chemoradiotherapy in postoperative or preoperative setting to guarantee the early administration of systemic chemotherapy in patients with elevated ESR level. Radiation Therapy Oncology Group (RTOG) 9514, a multi-institutional phase II trial for the evaluation of neoadjuvant chemotherapy and interdigitated RT in the management of large, high-grade STS, demonstrated high rate of disease control (5-year DFS, 56.1%; OS, 71.2%) but regrettably high severe toxicity (fatal grade 5, 5%; grade 4, 83%) [36, 37]. Recently, Chowdhary et al. reported the treatment outcomes of a modified RTOG 9514 regimen, without dacarbazine, and with modern RT techniques to reduce treatment-related toxicity [38]. The authors demonstrated that the omission of dacarbazine did not negatively impact survival outcomes (5-year DFS, 64%; OS, 81.2%) and there was no fatal grade 5 toxicity. If we select appropriate condition, more aggressive adjuvant treatment could be beneficial in high-risk group with elevated ESR level.

There were several limitations to our current study of note. First, the higher level of inflammatory markers is non-specific and may be associated with conditions other than STS. Although the ESR level was an independent prognostic factor after adjustment for other established prognostic factors, other chronic inflammatory conditions cannot be excluded. Second, although most of our patients (61.7%) had MFH or myxoid liposarcoma, our cohort included diverse histologic subtypes. The histologic subtype has been identified as a prognostic variable for STS outcomes. Finally, the retrospective design of our analysis may have led to a selection bias, and a relatively small number of patients were included. However, our report was strengthened by its use of a relatively homogenous study population that received surgery and postoperative RT for localized extremity STS at a single center.

## Conclusions

The preoperative serum ESR appears to represent an independent prognostic biomarker of OS in extremity STS patients, even after adjustment for known prognostic factors. This result suggests that preoperative assessment of the ESR, a readily available test, might improve the objective estimate of high-risk patients of tumor relapse. Further studies of a larger group of patients are required to confirm this strong link between the ESR and survival outcomes in STS.

## Data Availability

Please contact author for data requests.

## Abbreviations

CRP: C-reactive protein
CXT: Chemotherapy
ESR: Erythrocyte sedimentation rate
FNCLCC: Fédération Nationale des Centres de Lutte Contre le Cancer
MFH: Malignant fibrous histiocytoma
NLR: Neutrophil to lymphocyte ratio
RT: Radiotherapy
STS: Soft tissue sarcoma
